# The neurocognitive profile of post‐traumatic stress disorder (PTSD), major depressive disorder (MDD), and PTSD with comorbid MDD

**DOI:** 10.1002/brb3.1950

**Published:** 2021-03-05

**Authors:** Sheri‐Michelle Koopowitz, Karen Thea Maré, Heather J. Zar, Dan J. Stein, Jonathan C. Ipser

**Affiliations:** ^1^ Department of Psychiatry & Neuroscience Institute Faculty of Health Sciences University of Cape Town Cape Town South Africa; ^2^ Unit on Child & Adolescent Health South African Medical Research Council (SAMRC) Cape Town South Africa; ^3^ Department of Paediatrics & Child Health Red Cross War Memorial Children’s Hospital University of Cape Town Cape Town South Africa; ^4^ Unit on Risk and Resilience in Mental Disorders South African Medical Research Council (SAMRC) Cape Town South Africa

**Keywords:** cognition, cognitive dysfunction, comorbidity, depression, PTSD

## Abstract

**Objective:**

Neurocognitive dysfunction has been associated with post‐traumatic stress disorder (PTSD) and major depressive disorder (MDD). However, although PTSD is often comorbid with MDD, there is little neurocognitive work to date on individuals who suffer from both PTSD and MDD. Here, we compared neurocognitive domains in individuals with PTSD, MDD, and comorbid PTSD and MDD with those of healthy controls.

**Methods:**

Participants comprised of mothers enrolled in the Drakenstein Child Health Study, a study exploring child health determinants in the Drakenstein district, Western Cape. *N* = 175 mothers (between 18 and 50 years) were recruited and divided into 4 groups: PTSD, MDD, PTSD with MDD, and healthy controls. Participants were assessed using the computerized NIH Toolbox, and paper and pencil neurocognitive tests. Domains assessed included executive function, memory, attention, learning, and processing speed.

**Results:**

Distinct patterns of neurocognitive dysfunction were observed in this sample. PTSD was associated with more intrusion errors and MDD was associated with delayed recall impairment, relative to healthy controls. PTSD with comorbid MDD was associated with processing speed impairments, relative to healthy controls, and monodiagnostic groups. No group differences were observed on measures of attention and executive function.

**Conclusion:**

Distinct patterns of neurocognitive dysfunction were associated with diagnoses of MDD and PTSD. Greater anticipated dysfunction and impairment in comorbid PTSD and MDD was not observed, however. Further work is needed to replicate and extend these findings.

## Graphical abstract

Neurocognitive dysfunction of PTSD, depression, and PTSD with comorbid depression was examined in a group of low–middle‐income women. We found that PTSD, MDD, and PTSD with comorbid MDD presented with dysfunction in different cognitive domains.

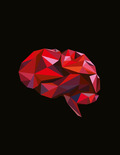

## 1 SIGNIFICANT OUTCOMES

- Post‐traumatic stress disorder (PTSD) was associated with greater intrusion errors.
- Major depressive disorder (MDD) was associated with impaired delayed recall.
- PTSD with comorbid MDD was associated with processing speed impairments.

## 2 LIMITATIONS

- All‐female sample was utilized.
- Both current and lifetime diagnoses included in clinical groups; therefore, state/strait cannot easily be differentiated.
- NIH Toolbox was administered in English and not participants' home language.
- Data for depression symptom severity were not collected.

## 3 INTRODUCTION

Post‐traumatic stress disorder (PTSD) is a trauma‐ and stressor‐related disorder that may develop after exposure to an event that is perceived to be a threat to an individual's body and/or mortality (American Psychiatric Association, 2013). In addition to experiencing a traumatic event, patients with PTSD also experience a variety of symptoms, such as intrusive re‐experiencing of the traumatic event, unintentional and intentional recall of the event, and distress caused by the event and the intrusive re‐experiencing (Ehlers et al., 2004).

Post‐traumatic stress disorder and Major depressive disorder (MDD) are highly comorbid disorders, although exact prevalence estimates vary between samples (Brady et al., 2000; Kaufman & Charney, 2000; O'Campo et al., 2006). For example, the National Comorbidity Survey, conducted in USA, reported that of the 10.4% of women who presented with lifetime PTSD, 48.5% also presented with comorbid lifetime MDD (Kessler et al., 1995). In community samples, PTSD with comorbid MDD is also common among female participants (Horesh et al., 2017; Rytwinski et al., 2013; Stein & Kennedy, 2001).

Examining the effects of comorbidity is important for a number of reasons. First, there is evidence that patients with comorbid PTSD and MDD may experience greater functional impairment than patients diagnosed with one of these disorders (Blanchard et al., 1998; Nijdam et al., 2013). Second, patients with comorbid PTSD and MDD may be less likely to respond to treatment and are less likely to remit than PTSD patients (Blanchard et al., 1998; Campbell et al., 2007; Flory & Yehuda, 2015). Third, while examining any mental disorder in isolation may yield useful insights, the investigation of the mechanisms involved in comorbidity may ultimately further contribute to our understanding of individual conditions.

Post‐traumatic stress disorder and MDD have both been reported in a number of peer‐reviewed studies to be associated with impairments in a number of neurocognitive domains. For example, mild to moderate executive functioning impairments have been observed in the patient groups, relative to both healthy and trauma‐exposed controls (Polak et al., 2012; Scott et al., 2015; Stricker et al., 2015). In PTSD, studies implicate deficits in multiple aspects of executive function, such as working memory, inhibition, flexibility, and set‐switching relative to trauma‐exposed and trauma naïve controls (Olff et al., 2014; Polak et al., 2012; Stein et al., 2002; Stricker et al., 2015). Similarly, in MDD participants relative to healthy, nonclinical controls, executive function impairments include deficits in working memory, set‐switching, and inhibition (Bora et al., 2013; Gohier et al., 2009; McIntyre et al., 2013; Snyder, 2013). In a systematic review of 18 neurocognitive PTSD studies, Polak et al. (2012) reported that patients with comorbid depression symptoms exhibited greater executive dysfunction, relative to patients without comorbid depression symptoms. The current literature suggests that PTSD is associated with sustained and divided attention impairments relative to healthy controls and controls exposed to trauma (Jenkins et al., 2000; Vasterling et al., 1998). Moderate deficits in general attention has been reported in MDD patients, as well as sustained and divided attention impairments, relative to healthy, nonclinical controls (Godard et al., 2012; Lee et al., 2012; Lim et al., 2013; Rock et al., 2014). Scheiner et al. (2014) reported that participants with PTSD and comorbid MDD performed comparably with all the groups and within the normal functioning range on measures of attention. Memory deficits have consistently been reported for both PTSD and MDD, individually (Lim et al., 2013; McIntyre et al., 2013; Rock et al., 2014; Scott et al., 2015). For example, in PTSD, the largest effects and impairments were observed for visual memory, while verbal memory and impaired verbal learning was also significantly impaired, relative to trauma‐exposed and healthy controls (Scott et al., 2015). In MDD, in addition to overall memory impairment, the largest effect was found for verbal memory (Lim et al., 2013; McIntyre et al., 2013; Rock et al., 2014). In one of a handful of comorbid samples, PTSD with comorbid MDD participants exhibited memory impairments, such as significant verbal learning deficits, poor short‐term cued recall, and impaired long‐term free recall (Nijdam et al., 2013). Finally, moderate processing speed impairments have consistently been reported in PTSD patients (Scott et al., 2015; Stricker et al., 2015; Twamley et al., 2009), as well as MDD patients (Lee et al., 2012; Lim et al., 2013; McDermott & Ebmeier, 2009; Snyder, 2013), relative to healthy controls.

Nevertheless, despite multiple reports of neurocognitive dysfunction in both MDD and PTSD in isolation, there is a lack of research examining the neurocognitive correlates of PTSD with comorbid MDD, despite the comorbid prevalence of these disorders. Therefore, it is not clear whether PTSD with comorbid MDD is associated with more severe impairment, or a qualitatively distinctive pattern of neurocognitive deficits, relative to PTSD and MDD individually.

### 3.1 Aims of this study

The main aim of this study was to determine whether a diagnosis of PTSD, MDD, and PTSD with comorbid MDD was associated with neurocognitive dysfunction in a sample of mothers in a low–middle‐income region of the Western Cape. We were interested in both differences in the severity of neurocognitive dysfunction between these groups, as well as variability in the pattern of impairment across neurocognitive domains. While there is insufficient evidence to support hypotheses regarding qualitative differences in function between groups, based on the literature we predicted that PTSD with comorbid MDD would be associated with greater cognitive dysfunction, relative to the monodiagnostic groups.

## 4 METHODS AND MATERIALS

### 4.1 Participants

Participants were recruited from the Drakenstein Child Health Study (DCHS) (Zar et al., 2014). Pregnant women were recruited from two primary healthcare clinics; Mbekweni (serving a predominantly black African community) and TC Newman (serving a mixed ancestry community). Mothers were enrolled in the Drakenstein Child Health Study at 20–28 weeks' gestation while attending routine antenatal care and were prospectively followed. Women were eligible for the study if they were 18 years or older, between 20 and 28 weeks' gestation, planned attendance at one of the two recruitment clinics and intended to remain in the area (Zar et al., 2014). Exclusion criteria for the present study included: (a) loss of consciousness longer than 30 min, (b) inability to speak English, (c) current/lifetime alcohol and/or substance dependence or abuse, (d) psychiatric illness, including psychosis, other than PTSD and/or MDD, and (e) traumatic brain injury. Psychological and physical trauma exposures were not exclusion criteria for the control group.

### 4.2 Ethics and ethical approval

This study was conducted in accordance with the Declaration of Helsinki. The DCHS was approved by the Faculty of Health Sciences, Human Research Ethics Committee, University of Cape Town (401/2009) and by the Western Cape Provincial Health Research committee. Mothers provided informed consent at enrollment and were reconsented annually. Consent was done in the mother's preferred language: English, Afrikaans, or isiXhosa.

### 4.3 Materials/Measures

#### 4.3.1 Diagnoses

All psychiatric interviews were conducted by a qualified clinician. The 5th edition of the Mini International Neuropsychiatric Interview (MINI) (Sheehan et al., 1998), which utilizes DSM‐IV diagnostic criteria, was used to determine whether participants presented with psychopathology. Women who had a current/lifetime PTSD diagnosis formed the PTSD group, participants who had a current/lifetime MDD diagnosis were part of the MDD group, and participants who had current/lifetime PTSD and MDD formed the comorbid group. Additionally, all cases of current and lifetime depression (including postpartum depression) were included. The control group consisted of participants from the DCHS with no current or lifetime psychiatric illness.

### 4.4 Neurocognitive assessment

#### 4.4.1 NIH Toolbox

The NIH Toolbox Cognition battery was used as the primary neurocognitive battery, where measures assessing memory, attention, processing speed, and executive function were used, as well as two measures described as “supplementary” (Weintraub et al., 2013). The measures that were utilized in this study included the Dimensional Change Card Sort test (executive function/set‐shifting), Flanker Control and Inhibition test (attention/executive function), the List Sorting Working Memory test (working memory), and the Pattern Comparison Processing Speed test (processing speed) (Weintraub et al., 2013). The supplementary measures included in this study were the Rey Auditory Verbal Learning test (memory/learning) and the Oral Symbol Digit test (processing speed). Although all tests were administered in English, tests that do not depend on verbal ability were given preference in the interests of cross‐cultural comparability, as the NIH Toolbox has not yet been translated into the home languages of participants in the Drakenstein Child Health Study.

#### 4.4.2 Additional neurocognitive measures

To date, no studies have been published using the NIH Toolbox within a South African context. Accordingly, a number of validated paper and pencil tests were included in the assessment in order to validate the NIH Toolbox results. The Wide Range Assessment of Memory and Learning II (WRAML II) (Sheslow & Adams, 1990) was used as a measure of immediate and delayed memory recall. The Color Trails Test 1 and 2 (D'Elia & Satz, 1996) were used to assess psychomotor processing speed and executive functioning, respectively. The Color Trails Test 1 was used as a measure of psychomotor speed, while the Color Trails Test 2 was used as a measure of task‐switching (Diamond, 2013; Porter et al., 2007). The Color Trails Test is ideal in the current research setting as it was developed to be free from cultural bias, and it can be administered nonverbally (D'Elia & Satz, 1996). The Color Trails Test includes an interference index (determined by subtracting Color Trails 1 time from Color Trails 2 time, then dividing by Color Trails 1 time), which helps obtain a purer measure of executive function by controlling for processing speed. The digit span forwards was used as a measure of attention, and the digit span backwards was used as a measure of working memory/executive function (Wechsler, 1997). These additional measures have been used successfully in a South African research context on numerous occasions (Gouse et al., 2012; Joska et al., 2011; Lipinska, 2017; Schoeman et al., 2009).

### 4.5 Procedure

All participants were assessed at the 18‐month postnatal DCHS visit at their primary health clinic. Neurocognitive testing typically took place in the morning, in a quiet room at the clinic. Participants began their assessment session by completing the paper and pencil tests (the WRAML II, Color Trails 1 and 2, and digit span, in that order). After these measures were completed, participants began the NIH Toolbox assessment. The NIH Toolbox was presented to the participant on a computer monitor, and participants were required to respond by pressing either the left or right arrow key on a keyboard. All task instructions were explained to the participants in English, and they were allowed to ask questions before commencing the assessment. As per NIH Toolbox protocol, a practice example was given to the participants for each test prior to commencement of testing. After the example, the participant performed the task with no additional help or guidance. In totality, each neurocognitive assessment was approximately one hour long.

### 4.6 Data analysis

As a preliminary analysis, bivariate correlation tests were conducted to examine associations between performance on the paper and pencil tests and the NIH Toolbox measures. Convergent validity was assessed by computing correlation coefficients between the Toolbox measures and paper and pencil tests that test the same domains (see Table 1). Discriminant validity of the toolbox measures was assessed by computing correlations between Toolbox measures and paper and pencil tests of different domains. Analysis of covariance tests (ANCOVA) was used to compare neurocognitive performance on the NIH Toolbox measures between groups. Age and years of education were included as covariates in the models. Analyses were conducted using raw scores for all tests, except for two measures, the Dimensional Change Card Sort, and the Flanker Attention and Inhibition measure. Both of these tests generate a final score which combines the participant's accuracy and reaction time to create a computed score (Slotkin et al., 2012). Interaction terms for group and age, as well as group and education, were only included in the final model if models where these interactions were tested separately as predictors of test scores were statistically significant (at alpha <0.1). This procedure was employed to maximize the power of the final models run to detect differences in test performance between groups. Where interactions were significant, correlations were run between the test score and the significant covariate within each group, to aid interpretation of findings.

**TABLE 1:** Measures used and their associated cognitive domains

Measure	Domain
NIH Toolbox	
Rey Auditory Verbal Learning Test	Verbal learning
Dimensional Change Card Sort	Executive function (set‐switching)
Flanker Inhibition and Control	Attention/inhibition
List Sorting Working Memory	Working memory
Oral Symbol Digit	Processing speed
Pattern Comparison Processing Speed	Processing speed
Paper and Pencil tests	
WRAML II immediate recall	Verbal learning/memory
WARML II delayed recall	Delayed recall/memory
Color Trails Test 1	Processing speed
Color Trails Test 2	Executive function (set‐switching)
Digit Span Forwards	Attention
Digit Span Backwards	Working memory

#### 4.6.1 Executive function composite score

As a number of executive function tasks were utilized in this study, an executive function composite score was created to determine associations between diagnoses of PTSD, MDD, and PTSD with comorbid MDD and global executive function. The composite score was created by averaging scaled scores for each of the executive function measures, including the Dimensional Change Card Sort (set‐shifting), Flanker Attention and Inhibition (inhibition), List Sorting Working Memory (working memory), Color Trails Test 2 (set‐shifting), and digit span backwards (working memory).

## 5 RESULTS

The total sample (*n* = 175) was made up as follows: PTSD group *n* = 36 (current PTSD *n* = 3); MDD group *n* = 30 (current MDD *n* = 2); PTSD + MDD group *n* = 23 (current PTSD *n* = 1; current MDD *n* = 3); and control group *n* = 86.

Demographic variables for the sample are presented in Table 2. There were no significant differences between the four groups on age (*F*(3, 171) = 1.74, *p* = .162). There was, however, a significant difference between groups on education level (*F*(3, 171) = 4.04, *p* = .008). Post hoc comparisons (Tukey's) indicated that the average level of education was significantly higher in the control group, relative to the comorbid group (*p* = .009). The effect size, calculated using eta squared, was 0.06, a moderate effect size (Rosenthal & Rosnow, 2008). The PTSD, MDD, and control groups were comparable with respect to level of education. There was a significant difference between the PTSD group and the comorbid group on PTSD symptom severity with the comorbid group exhibiting more severe PTSD symptoms (*t*(57) = −3.24, *p* = .002). The participants experienced a variety of trauma types, including physical assault (16.6%), sexual assault (16%), weapon assault (8.6%), and transport accidents (4%).

**TABLE 2:** Demographic data

	PTSD	MDD	PTSD + MDD	Control	*F/t*	*p*
	*n* = 36	*n* = 30	*n* = 23	*n* = 86		
Age mean (SD)	28.86 (6.56)	28.17 (6.24)	30.7 (6.92)	27.56 (5.51)	1.73	.16
Education mean^a^ (SD)	10.78 (2.76)	10.83 (2.32)	9.96 (2.4)	11.65 (1.96)	4.04	.008
CAPS total score (SD)	70.44 (17.44)	‐	85.87 (18.49)	‐	−3.24	.002
Site (Mbe:TC)	21:15	11:19	9:14	50:36		

^a^ Education measured in years of formal schooling.

### 5.1 NIH Toolbox validity

Bivariate correlation coefficients were computed between the NIH Toolbox task scores (RAVLT, DCCS, Flanker Inhibition and Control, LSWM, Oral Symbol Digit, and Pattern Comparison Processing Speed test) and the paper and pencil test scores (WRAML II, CT 1 and 2, and digit span forwards and backwards). A threshold of *p* < .01 was utilized for determination of the convergent and divergent validity. The results indicate that there are small to medium correlations between the NIH Toolbox measures and the paper and pencil tests (Table 3). Moreover, the observation that moderate to strong correlations(Cohen, 1988) were observed between scores for tests of the same domain (e.g., RAVLT and WRAML II) provides confidence in the validity of the NIH Toolbox measures for this particular research setting (see Table 4).

**TABLE 3:** NIH Toolbox correlations with paper and pencil tests

NIH Toolbox measures	Paper and pencil tests		CT 2	Digit F	Digit B
	WRAML total	CT 1			
DCCS	.42**	−.3**	−.53**	.32**	.29**
Flanker inhibition	.24**	−.24**	−.49**	.25**	.17*
LSWM	.36**	−.3**	−.5**	.36**	.28**
RAVLT	.61**	−.23**	−.3**	.33**	.28**
Oral symbol digit	.39**	−.39**	−.59**	.29**	.23**
Pattern comparison	.31**	−.38**	−.49**	.22**	.1

* Significant at *p* = .05.

** Significant at *p* < .001.

**TABLE 4:** Average correlation coefficients of tests that measure the same construct

Measures	Average correlation
DCCS and Color Trails 2	−.53**
Flanker Inhibition and Digit Span Forwards	.25**
LSWM and Digit Span Backwards	.28**
RAVLT and WRAML II	.61**
Oral Symbol digit and Color Trails 1	−.39**
Pattern Comparison	−.38**

** Significant at *p* < .001.

### 5.2 Neurocognitive function results

Results from the ANCOVA tests can be found in Table 5.

**TABLE 5:** Means, standard deviations, and ANCOVA test statistics for neurocognitive tests

	PTSD	MDD	PTSD + MDD	Control	*F*	*p*
	*n* = 36	*n* = 30	*n* = 23	*n* = 86		
NIH Toolbox measures						
DCCS	−0.14 (1.12)	0.05 (1.23)	−0.39 (1.1)	0.15 (0.79)	0.45	.72
List sorting WM	13.81 (3.34)	14.34 (3.42)	13.09 (3.54)	14.69 (2.9)	2.29	.08
Flanker	6.82 (1.17)	6.78 (1.24)	6.44 (1.14)	6.97 (1.02)	0.18	.91
RAVLT	19.44 (6.79)	19.93 (5.32)	17.48 (4.9)	22.38 (5.13)	2.13	.09
Oral symbol digit	62.24 (17.13)	58.07 (14.83)	54.7 (14.52)	63.69 (14.72)	1.03	.38
Pattern comparison	39.14 (12.12)	41.03 (10.63)	38.74 (14.16)	41.6 (11.21)	3.02	.032* ^a^
Additional measures						
Color Trails 2 time	122.19 (32.94)	116.62 (33.03)	127.95 (43)	118.62 (36.8)	0.23	.88
CT interference index	0.65 (0.57)	0.68 (0.57)	0.82 (0.5)	0.73 (0.57)	0.47	.71
Digit span backwards	4.09 (1.49)	4.21 (1.42)	3.77 (1.44)	4.16 (1.59)	0.23	.88
Digit span forwards	7.34 (1.86)	7.17 (2.12)	6.95 (1.36)	7.5 (1.66)	2.38	.07
WRAML total score	28.78 (9.99)	26.83 (7.9)	26.14 (6.91)	30.15 (7.18)	1.97	.12
WRAML intrusions	3.31 (2.75)	2.31 (2.28)	2.36 (1.92)	1.85 (1.72)	3.46	.018* ^b^
WRAML delayed recall	8.28 (3.29)	6.69 (2.36)	7.05 (2.65)	8.5 (2.54)	2.72	.046* ^c^
Color Trails 1 time	4.31 (0.29)	4.24 (0.24)	4.24 (0.27)	4.23 (0.35)	0.42	.74
Composite score						
EF composite	−0.018 (0.69)	−0.029 (0.69)	−0.317 (0.73)	0.105 (0.59)	0.54	.65

^a^ No significant pairwise differences observed;

^b^ PTSD exhibited significantly more intrusions than controls (*p* = .002), and PTSD exhibited more intrusions than MDD (*p* = .051);

^c^ MDD significantly worse recall than controls (*p* = .01), and MDD significantly worse recall than PTSD (*p* = .017).

* *p* < .05.

### 5.3 Executive function measures

#### 5.3.1 NIH Toolbox

##### Dimensional Change Card Sort test (DCCS)

There was no significant interaction between the covariates and the independent variable; therefore, the interaction terms were removed from the model. The ANCOVA test showed that there was no significant group effect, *F*(3, 168) = 0.45, *p* = .72. Across all groups, greater age was associated with worse performance and more education was associated with better performance (at *p* < .001). The final model explained almost 20% of the variability in test scores (adjusted *R* ^2^ = .186).

##### List Sorting Working Memory test (LSWM)

Both covariates produced significant interaction terms and remained in the model. The comorbid group displayed a significant negative correlation between age and test performance (*r* = −.55; *p* = .003). In contrast, higher levels of education were associated with better performance on the LSWM only for PTSD (*r* = .52; *p* < .001) and MDD groups (*r* = .35; *p* = .03), and not for the comorbid group. A relatively small portion of the variability (10.6%) in test scores were explained by the final model (adjusted *R* ^2^ = .106).

#### 5.3.2 Paper and pencil test

##### Color Trails 2 (CT 2)

There were no significant interaction terms for the covariates; therefore, the interaction terms were removed from the final model. There was no significant group effect in the final model, *F*(3, 163) = 0.23, *p* = .61, and this model explained roughly 10% of the variability in test scores (adjusted *R* ^2^ = .098).

##### Digit Span Backwards

The covariates did not produce significant interaction terms and were removed from the final model. The final ANCOVA indicated that there was no significant group effect, *F*(3, 163) = 0.23, *p* = .42, and less than 10% of the variability in test scores were explained by the final model (adjusted *R* ^2^ = .08).

#### 5.3.3 Executive function composite score

There were no significant interaction terms for age and education and were therefore removed from the final model. The final model showed that there was no significant group effect, *F*(3, 162) = 0.54, *p* = .216, and approximately a fifth of the variability in the test scores (adjusted *R* ^2^ = .206) was explained by the final model.

### 5.4 Attention

#### 5.4.1 NIH Toolbox

##### Flanker Inhibition and Control

There were no significant interactions for age and education, so the interaction terms were removed from the model. The final ANCOVA model showed that there was no significant group effect on this measure, *F*(3, 169) = 0.18, *p* = .91, and approximately 18% of the variability in test scores is explained by the final model (adjusted *R* ^2^ = .175).

#### 5.4.2 Paper and pencil tests

##### Digit Span forwards

Age, but not education, was retained in the final model (*F*(3, 160) = 0.2.61, *p* = .054). There was a significant negative correlation between age and test score for the PTSD group (*r* = −.41, *p* = .011), showing that increased age led to poorer digit span forwards results in the PTSD group. No association was found for the other groups. The final model explained approximately 7% of the variance in test scores (adjusted *R* ^2^ = .072).

### 5.5 Memory and learning

#### 5.5.1 NIH Toolbox

##### Rey Auditory Verbal Learning Test (RAVLT)

Both age (*F*(3, 165) = 2.32, *p* = .077) and education (*F*(3, 165) = 2.86, *p* = .039) produced significant interaction terms, and these terms were kept in the model. Older age was negatively associated with performance on the RAVLT, but only in participants with PTSD (PTSD: *r* = −.32, *p* = .029; PTSD + MDD: *r* = −.54; *p* = .004). When examining the correlations between education level and RAVLT scores, the PTSD group showed the strongest positive correlation (*r* = .61, *p* < .001), followed by the MDD group (*r* = .39, *p* = .018), then the control group (*r* = .21, *p* = .027). The comorbid group did not show a significant correlation. The final model explained less than a quarter of the variability of the test scores (adjusted *R* ^2^ = .223).

#### 5.5.2 Paper and pencil tests

##### WRAML II total

The interaction term for education was significant and remained in the final model, *F*(3, 160) = 2.17, *p* = .094. The correlation between education level and WRAML II total score was positive and significant for the PTSD group (*r* = .66, *p* < .001), comorbid group (*r* = .43, *p* = .023), and the control group (*r* = .26, *p* = .008), but not for the MDD group (*r* = .22, *p* = .124). The final model explained 19% of the test score variance (adjusted *R* ^2^ = .19).

##### WRAML II delayed recall

Neither age nor education produced significant interaction terms and was removed from the final model. The final ANCOVA model indicated that there was a significant group effect, *F*(3, 163) = 2.72, *p* = .046, and approximately 17% of the variance in test scores was explained by the final model (adjusted *R* ^2^ = .175). Significant pairwise group differences were found between the control group and the MDD group (*p* = .01) and between the MDD group and the PTSD group (*p* = .017), with the MDD group exhibiting significantly fewer recalled words than the controls and PTSD group, respectively. Comparable test performance was observed when comparing the control group and either of the PTSD groups (PTSD, comorbid).

##### WRAML II intrusions

Interaction terms for age and education were not significant; therefore, they were removed from the model. The final ANCOVA model found a significant group effect, *F*(3, 163) = 3.46, *p* = .018. The final model explained almost 6% of the total variance in the intrusions test scores (adjusted *R* ^2^ = .058). Significant pairwise group differences were found between the control group and PTSD group (*p* = .002) with the PTSD group exhibiting significantly more incorrectly recalled words than the control group. A pairwise comparison between the MDD group and PTSD group revealed differences that approached significance (*p* = .051), with the PTSD group demonstrating more incorrectly recalled words than the MDD group.

### 5.6 Processing speed measures

#### 5.6.1 NIH Toolbox

##### Oral Symbol Digit test

The covariates did not produce significant interaction terms; thus, the interaction terms were not included in the final model. The final model indicated that there was no significant group effect, *F*(3, 167) = 1.03, *p* = .38, and approximately a quarter of the variance of test scores was explained by the final model (adjusted *R* ^2^ = .24).

##### Pattern Comparison Processing Speed test

Age did not produce a significant interaction term and was therefore left out of the model. A significant education interaction effect was observed (*F*(3, 166) = 2.86, *p* = .038). A significant group effect was observed in the final model, *F*(3, 166) = 3.01, *p* = .032 with the PTSD and PTSD with comorbid group performing worse than controls and the MDD group. Post hoc analysis revealed that level of education was significantly and positively correlated with test score for the PTSD group (*r* = .51, *p* < .001) and the controls (*r* = .4, *p* < .001). The final model explained almost a quarter of the variability of test scores (adjusted *R* ^2^ = .22).

#### 5.6.2 Paper and pencil tests

##### Color Trails 1 (CT1)

Age and education did not produce significant interaction terms and the interaction terms were removed from the final model. The final ANCOVA model showed that there was no significant group effect, *F*(3, 163) = 0.42, *p* = .388, with only a tiny portion of the variability in test scores explained by this model (adjusted *R* ^2^ = .009).

## 6 DISCUSSION

In this study, PTSD was associated with more incorrectly recalled words, relative to controls, while MDD was associated with delayed recall impairments; both findings were obtained using the WRAML II battery. PTSD with comorbid MDD was associated with processing speed impairments which were observed on the NIH Toolbox Pattern Comparison Processing Speed Test.

Our finding that PTSD was associated with more incorrectly recalled words, or intrusion errors, suggests the presence of disinhibition and attentional deficits (Lezak et al., 2004). This finding could translate into incorrectly recalling instructions (or, e.g., recalling instructions that were not given) in a patient's daily life. These instances could have a negative effect on daily living. However, attention deficits were not observed on other measures of attention in this group. To fully understand the implications of this work, further work using more detailed neurocognitive tests of sustained and divided attention would be useful.

Although MDD tends to be associated with global memory dysfunction (Lee et al., 2012; Lim et al., 2013), we found that the MDD was associated with specific memory dysfunction rather than global dysfunction. Thus, delayed recall impairments (but not immediate recall impairments) were observed for the MDD group, relative to the control group. This finding suggests that MDD patients may struggle to consolidate memories, which would negatively impact instances requiring memorization and consolidation of information.

Post‐traumatic stress disorder with comorbid MDD was associated with poorer and slower processing speed on the Pattern Comparison processing speed test, relative to monodiagnostic groups and healthy controls. However, no group effect was observed on the other processing speed measures. To our knowledge, processing speed in participants with PTSD and comorbid MDD has not previously been assessed or reported in the literature. This finding is important insofar as it may contribute toward functional impairment in daily life. For example, patients with PTSD and comorbid MDD may be more at risk, due to slower processing speed, in assessing a risky situation in which they need to make snap decisions.

Exploring the interactions of age and education on group differences indicated that relative to controls, older age was associated with worse performance on measures of learning (RAVLT), attention (digit span forwards), and executive function (LSWM) in participants diagnosed with PTSD (PTSD and comorbid groups). No effect of age was found for the MDD group on these tasks. Further, education tends to have a protective effect on the monodiagnostic groups on the RAVLT. Higher levels of education were associated with better working memory performance, as assessed using the LSWM, for the monodiagnostic groups, but not the comorbid group, compared with controls. This finding suggests that the protective effect of education is reduced for the comorbid group.

In our sample, very specific neurocognitive impairments were observed in the clinical groups, rather than general neurocognitive dysfunction, or cognitive dysfunction across all domains. Our findings suggest that PTSD with comorbid MDD is not necessarily associated with greater neurocognitive impairment across all domains, relative to the monodiagnostic groups and controls. Rather, different impairments were observed for the comorbid group. Furthermore, this study found that there were no significant differences between any of the groups on executive functioning measures such as the Color Trails Tests, digit span, and the working memory measure (List Sorting Working Memory).

One possible reason that may contribute to differences between our findings on executive function and attentional impairments and those previously reported is a sampling difference. The present study utilized a sample that is different from the majority of published PTSD studies. For example, women from a LMI region who have experienced a variety of trauma types (predominantly physical assault, sexual assault, weapon assault, and transport accidents) were utilized in the present study, whereas the majority of PTSD‐based studies utilize male veteran samples, particularly from the United States of America. However, Stein and colleagues employed a female‐only sample with high levels of interpersonal violence (IPV)‐related trauma and report similar findings with the PTSD group and control group performing comparably on measures such as Trail Making Test, the digit span, and measures of memory (Stein et al., 2002). Relatively small effect sizes have also found for neurocognitive impairment in female combat veterans compared with males (Johnsen & Asbjørnsen, 2008).

## 7 CONCLUSION

In summary, we found distinct patterns of neurocognitive dysfunction were associated with diagnoses of PTSD and MDD. However, greater anticipated impairment in comorbid PTSD and MDD was not observed. Given the limitations of this work, further research with larger sample sizes is needed to replicate and extend these findings.

## CONFLICT OF INTEREST

The authors report no conflicts of interest. This work was supported and funded by the Bill and Melinda Gates Foundation (OPP 1017641). Additional support for HJZ and DJS was provided by the South African Medical Research Council.

## AUTHORS' CONTRIBUTIONS

All authors contributed to this work equally. SK, HJZ, DJS, and JI: Conception and design of the study; SK and KTM: Data acquisition; SK and JI: Data analysis; SK, DJS, and JI: Data interpretation; SK: Write up; SK, KTM, JI, and DJS: Editing; All authors: Final approval.

### Peer Review

The peer review history for this article is available at https://publons.com/publon/10.1002/brb3.1950.

## Data Availability

The Drakenstein Child Health Study is committed to the principle of data sharing. Deidentified data will be made available to requesting researchers as appropriate. Requests for collaborations to undertake data analysis are welcome. More information can be found on our website [http://www.paediatrics.uct.ac.za/scah/dclhs].

## References

[brb31950-bib-0001] American Psychiatric Association (2013). Diagnostic and statistical manual of mental disorders (DSM‐5®). American Psychiatric Publishing.

[brb31950-bib-0002] Blanchard, E. B. , Buckley, T. C. , Hickling, E. J. , & Taylor, A. E. (1998). Posttraumatic stress disorder and comorbid major depression: Is the correlation an illusion? Journal of Anxiety Disorders, 12(1), 21–37. 10.1016/S0887-6185(97)00047-9 9549607

[brb31950-bib-0003] Bora, E. , Harrison, B. , Yücel, M. , & Pantelis, C. (2013). Cognitive impairment in euthymic major depressive disorder: A meta‐analysis. Psychological Medicine, 43(10), 2017–2026. 10.1017/S0033291712002085 23098294

[brb31950-bib-0004] Brady, K. T. , Killeen, T. K. , Brewerton, T. , & Lucerini, S. (2000). Comorbidity of psychiatric disorders and posttraumatic stress disorder. Journal of Clinical Psychiatry, 61, 22–32. 10795606

[brb31950-bib-0005] Campbell, D. G. , Felker, B. L. , Liu, C.‐F. , Yano, E. M. , Kirchner, J. A. E. , Chan, D. , Rubenstein, L. V. , & Chaney, E. F. (2007). Prevalence of depression–PTSD comorbidity: Implications for clinical practice guidelines and primary care‐based interventions. Journal of General Internal Medicine, 22(6), 711–718. 10.1007/s11606-006-0101-4 17503104 PMC2219856

[brb31950-bib-0006] Cohen, J. (1988). Statistical power analysis for the behavioral sciences, 2nd ed. Erihaum.

[brb31950-bib-0007] D'Elia, L. , & Satz, P. (1996). Color trails test. Psychological Assessment Resources.

[brb31950-bib-0008] Diamond, A. (2013). Executive functions. Annual Review of Psychology, 64, 135–168. 10.1146/annurev-psych-113011-143750 PMC4084861 23020641

[brb31950-bib-0009] Ehlers, A. , Hackmann, A. , & Michael, T. (2004). Intrusive re‐experiencing in post‐traumatic stress disorder: Phenomenology, theory, and therapy. Memory, 12(4), 403–415. 10.1080/09658210444000025 15487537

[brb31950-bib-0010] Flory, J. D. , & Yehuda, R. (2015). Comorbidity between post‐traumatic stress disorder and major depressive disorder: Alternative explanations and treatment considerations. Dialogues in Clinical Neuroscience, 17(2), 141. 26246789 10.31887/DCNS.2015.17.2/jflory PMC4518698

[brb31950-bib-0011] Godard, J. , Baruch, P. , Grondin, S. , & Lafleur, M. F. (2012). Psychosocial and neurocognitive functioning in unipolar and bipolar depression: A 12‐month prospective study. Psychiatry Research, 196(1), 145–153. 10.1016/j.psychres.2011.09.013 22370154

[brb31950-bib-0012] Gohier, B. , Ferracci, L. , Surguladze, S. A. , Lawrence, E. , El Hage, W. , Kefi, M. Z. , Allain, P. , Garre, J.‐B. , & Le Gall, D. (2009). Cognitive inhibition and working memory in unipolar depression. Journal of Affective Disorders, 116(1), 100–105. 10.1016/j.jad.2008.10.028 19042027

[brb31950-bib-0013] Gouse, H. , Thomas, K. G. , & Solms, M. (2012). Neuropsychological, functional, and behavioral outcome in South African traumatic brain injury litigants. Archives of Clinical Neuropsychology, 28(1), 38–51. 10.1093/arclin/acs100 23151324

[brb31950-bib-0014] Horesh, D. , Lowe, S. R. , Galea, S. , Aiello, A. E. , Uddin, M. , & Koenen, K. C. (2017). An in‐depth look into PTSD‐depression comorbidity: A longitudinal study of chronically‐exposed Detroit residents. Journal of Affective Disorders, 208, 653–661. 10.1016/j.jad.2016.08.053 27816322 PMC6684032

[brb31950-bib-0015] Jenkins, M. A. , Langlais, P. J. , Delis, D. , & Cohen, R. A. (2000). Attentional dysfunction associated with posttraumatic stress disorder among rape survivors. The Clinical Neuropsychologist, 14(1), 7–12. 10.1076/1385-4046(200002)14:1;1-8;FT007 10855055

[brb31950-bib-0016] Johnsen, G. E. , & Asbjørnsen, A. E. (2008). Consistent impaired verbal memory in PTSD: A meta‐analysis. Journal of Affective Disorders, 111(1), 74–82. 10.1016/j.jad.2008.02.007 18377999

[brb31950-bib-0017] Joska, J. A. , Westgarth‐Taylor, J. , Hoare, J. , Thomas, K. G. , Paul, R. , Myer, L. , & Stein, D. J. (2011). Validity of the international HIV dementia scale in South Africa. AIDS Patient Care and STDs, 25(2), 95–101. 10.1089/apc.2010.0292 21214343

[brb31950-bib-0018] Kaufman, J. , & Charney, D. (2000). Comorbidity of mood and anxiety disorders. Depression and Anxiety, 12(s 1), 69–76. 10.1002/1520-6394(2000)12:1+<69:AID-DA9>3.0.CO;2-K 11098417

[brb31950-bib-0019] Kessler, R. C. , Sonnega, A. , Bromet, E. , Hughes, M. , & Nelson, C. B. (1995). Posttraumatic stress disorder in the National Comorbidity Survey. Archives of General Psychiatry, 52(12), 1048–1060. 10.1001/archpsyc.1995.03950240066012 7492257

[brb31950-bib-0020] Lee, R. S. , Hermens, D. F. , Porter, M. A. , & Redoblado‐Hodge, M. A. (2012). A meta‐analysis of cognitive deficits in first‐episode major depressive disorder. Journal of Affective Disorders, 140(2), 113–124. 10.1016/j.jad.2011.10.023 22088608

[brb31950-bib-0021] Lezak, M. D. , Howieson, D. B. , Loring, D. W. , & Fischer, J. S. (2004). Neuropsychological assessment. Oxford University Press.

[brb31950-bib-0022] Lim, J. H. , Oh, I. K. , Han, C. , Huh, Y. J. , Jung, I.‐K. , Patkar, A. A. , Steffens, D. C. , & Jang, B.‐H. (2013). Sensitivity of cognitive tests in four cognitive domains in discriminating MDD patients from healthy controls: A meta‐analysis. International Psychogeriatrics, 25(9), 1543–1557. 10.1017/S1041610213000689 23725644

[brb31950-bib-0023] Lipinska, M. (2017). Associations between sleep and cognitive‐affective functioning in Posttraumatic Stress Disorder. University of Cape Town.

[brb31950-bib-0024] McDermott, L. M. , & Ebmeier, K. P. (2009). A meta‐analysis of depression severity and cognitive function. Journal of Affective Disorders, 119(1), 1–8. 10.1016/j.jad.2009.04.022 19428120

[brb31950-bib-0025] McIntyre, R. S. , Cha, D. S. , Soczynska, J. K. , Woldeyohannes, H. O. , Gallaugher, L. A. , Kudlow, P. , Alsuwaidan, M. , & Baskaran, A. (2013). Cognitive deficits and functional outcomes in major depressive disorder: Determinants, substrates, and treatment interventions. Depression and Anxiety, 30(6), 515–527. 10.1002/da.22063 23468126

[brb31950-bib-0026] Nijdam, M. J. , Gersons, B. P. , & Olff, M. (2013). The role of major depression in neurocognitive functioning in patients with posttraumatic stress disorder. European Journal of Psychotraumatology, 4(1), 19979. 10.3402/ejpt.v4i0.19979 PMC3644057 23671761

[brb31950-bib-0027] O'Campo, P. , Kub, J. , Woods, A. , Garza, M. , Jones, A. S. , Gielen, A. C. , Dienemann, J. , & Campbell, J. (2006). Depression, PTSD, and comorbidity related to intimate partner violence in civilian and military women. Brief Treatment and Crisis Intervention, 6(2), 99. 10.1093/brief-treatment/mhj010

[brb31950-bib-0028] Olff, M. , Polak, A. R. , Witteveen, A. B. , & Denys, D. (2014). Executive function in posttraumatic stress disorder (PTSD) and the influence of comorbid depression. Neurobiology of Learning and Memory, 112, 114–121. 10.1016/j.nlm.2014.01.003 24440596

[brb31950-bib-0029] Polak, A. R. , Witteveen, A. B. , Reitsma, J. B. , & Olff, M. (2012). The role of executive function in posttraumatic stress disorder: A systematic review. Journal of Affective Disorders, 141(1), 11–21. 10.1016/j.jad.2012.01.001 22310036

[brb31950-bib-0030] Porter, R. J. , Bourke, C. , & Gallagher, P. (2007). Neuropsychological impairment in major depression: Its nature, origin and clinical significance. Australian and New Zealand Journal of Psychiatry, 41(2), 115–128. 10.1080/00048670601109881 17464689

[brb31950-bib-0031] Rock, P. , Roiser, J. , Riedel, W. , & Blackwell, A. (2014). Cognitive impairment in depression: A systematic review and meta‐analysis. Psychological Medicine, 44(10), 2029–2040. 10.1017/S0033291713002535 24168753

[brb31950-bib-0032] Rosenthal, R. , & Rosnow, R. L. (2008). Essentials of behavioral research: Methods and data analysis, 3rd ed. McGraw Hill.

[brb31950-bib-0033] Rytwinski, N. K. , Scur, M. D. , Feeny, N. C. , & Youngstrom, E. A. (2013). The co‐occurrence of major depressive disorder among individuals with posttraumatic stress disorder: A meta‐analysis. Journal of Traumatic Stress, 26(3), 299–309. 10.1002/jts.21814 23696449

[brb31950-bib-0034] Scheiner, D. L. , Keilp, J. , Mindt, M. R. , Burke, A. K. , Oquendo, M. A. , & Mann, J. J. (2014). Verbal learning deficits in posttraumatic stress disorder and depression. Journal of Traumatic Stress, 27(3), 291–298. 10.1002/jts.21921 24850268

[brb31950-bib-0035] Schoeman, R. , Carey, P. , & Seedat, S. (2009). Trauma and posttraumatic stress disorder in South African adolescents: A case‐control study of cognitive deficits. The Journal of Nervous and Mental Disease, 197(4), 244–250. 10.1097/NMD.0b013e31819d9533 19363380

[brb31950-bib-0036] Scott, J. C. , Matt, G. E. , Wrocklage, K. M. , Crnich, C. , Jordan, J. , Southwick, S. M. , Krystal, J. H. , & Schweinsburg, B. C. (2015). A quantitative meta‐analysis of neurocognitive functioning in posttraumatic stress disorder. Psychological Bulletin, 141(1), 105. 10.1037/a0038039 25365762 PMC4293317

[brb31950-bib-0037] Sheehan, D. V. , Lecrubier, Y. , Sheehan, K. H. , Amorim, P. , Janavs, J. , Weiller, E. , & Dunbar, G. C. (1998). The Mini‐International Neuropsychiatric Interview (MINI): The development and validation of a structured diagnostic psychiatric interview for DSM‐IV and ICD‐10. Journal of Clinical Psychiatry, 59, 22–33. 9881538

[brb31950-bib-0038] Sheslow, D. , & Adams, W. (1990). Wide range assessment of memory and learning (WRAML). Wide Range Incorporated.

[brb31950-bib-0039] Slotkin, J. , Nowinski, C. , Hays, R. , Beaumont, J. , Griffith, J. , Magasi, S. , & Gershon, R. (2012). NIH Toolbox scoring and interpretation guide (pp. 6–7). National Institutes of Health.

[brb31950-bib-0040] Snyder, H. R. (2013). Major depressive disorder is associated with broad impairments on neuropsychological measures of executive function: A meta‐analysis and review. Psychological Bulletin, 139(1), 81. 10.1037/a0028727 22642228 PMC3436964

[brb31950-bib-0041] Stein, M. B. , & Kennedy, C. (2001). Major depressive and post‐traumatic stress disorder comorbidity in female victims of intimate partner violence. Journal of Affective Disorders, 66(2), 133–138. 10.1016/S0165-0327(00)00301-3 11578665

[brb31950-bib-0042] Stein, M. B. , Kennedy, C. M. , & Twamley, E. W. (2002). Neuropsychological function in female victims of intimate partner violence with and without posttraumatic stress disorder. Biological Psychiatry, 52(11), 1079–1088. 10.1016/S0006-3223(02)01414-2 12460691

[brb31950-bib-0043] Stricker, N. H. , Keller, J. E. , Castillo, D. T. , & Haaland, K. Y. (2015). The neurocognitive performance of female veterans with posttraumatic stress disorder. Journal of Traumatic Stress, 28(2), 102–109. 10.1002/jts.22000 25847622

[brb31950-bib-0044] Twamley, E. W. , Allard, C. B. , Thorp, S. R. , Norman, S. B. , Hami cissell, S. , Hughes berardi, K. , Grimes, E. M. , & Stein, M. B. (2009). Cognitive impairment and functioning in PTSD related to intimate partner violence. Journal of the International Neuropsychological Society, 15(6), 879–887. 10.1017/S135561770999049X 19703324

[brb31950-bib-0045] Vasterling, J. J. , Brailey, K. , Constans, J. I. , & Sutker, P. B. (1998). Attention and memory dysfunction in posttraumatic stress disorder. Neuropsychology, 12(1), 125. 10.1037/0894-4105.12.1.125 9460740

[brb31950-bib-0046] Wechsler, D. (1997). WAIS‐III: Wechsler adult intelligence scale. Psychological Corporation.

[brb31950-bib-0047] Weintraub, S. , Dikmen, S. S. , Heaton, R. K. , Tulsky, D. S. , Zelazo, P. D. , Bauer, P. J. , Carlozzi, N. E. , Slotkin, J. , Blitz, D. , Wallner‐Allen, K. , Fox, N. A. , Beaumont, J. L. , Mungas, D. , Nowinski, C. J. , Richler, J. , Deocampo, J. A. , Anderson, J. E. , Manly, J. J. , Borosh, B. , … Gershon, R. C. (2013). Cognition assessment using the NIH Toolbox. Neurology, 80(11 Supplement 3), S54–S64. 10.1212/WNL.0b013e3182872ded 23479546 PMC3662346

[brb31950-bib-0048] Zar, H. , Barnett, W. , Myer, L. , Stein, D. , & Nicol, M. (2014). Investigating the early‐life determinants of illness in Africa: the Drakenstein Child Health Study. Thorax, 70(6), 592–594. 25228292 10.1136/thoraxjnl-2014-206242 PMC5107608

